# Genes Matter!

**DOI:** 10.4103/0974-7753.66900

**Published:** 2010

**Authors:** Patrick Yesudian

**Affiliations:** President, The Hair Research Society of India, No. 10, Ritherdon Avenue, Vepery, Chennai - 600 007, India. Email: patnirmu@gmail.com

Francis Crick, who, along with Watson unraveled the mystery of the nuclear thread of life DNA, for which he got the Nobel Prize, said “Almost all aspects of life are engineered at the molecular level and without understanding molecules, we can only have a sketchy understanding of life itself.” He was of course referring to the genes.

Genes are not proteins. Genes are not enzymes. Genes are messages which carry the blueprint of life. At the turn of the century, the completion of the human genome sequence consisting of 3.2 billion base pairs was announced to the world by the combined efforts of several advanced countries. This raised awesome possibilities to humanity, mostly good, but a few bad, akin to the discovery of the atom by physicists. Interestingly, these base pairs are found in just about 30,000 genes, almost the same number of genes as found in the lowly round worm, *Ascaris lumbricoidalis* – a blow to the human ego!

The molecular basis of a large number of genetic hair disorders has been elucidated in the last two decades. Likewise, in many of the genodermatoses with hair anomalies, the responsible genes have been identified. Obviously, absence of hair does not take away life but it takes away the enjoyment of life. It can be distressing to a young patient and equally so to the parents. The rapidly expanding body of information at the genetic (molecular) level has brought about major changes in the life of families affected by these hair disorders by providing them with a definitive diagnosis, possibility of premarital counseling and prenatal diagnosis and hopefully innovative therapies including gene replacement in the not too distant future.

The recent discovery of a gene *Lhx2* which leads to more hair growth raises exciting possibilities of treating baldness in the next decade. This gene is active during the anagen and it is turned off during the telogen.

Even in conditions where environment and stress play a predominant role in causing baldness, these factors act through predisposed genes. An old dermatologist told a young man with androgenetic alopecia “As failed your father’s genes, so have yours.” So, genes matter and genetics has permeated into every nook and corner of the field of trichology.

That is why in the First International Congress of Trichology “Hair India 2010,” organized by the Hair Research Society of India, an entire session is dedicated to Genotrichology with distinguished pioneers in the field like Prof. Regina Betz who discovered the gene for hypotrichosis, delivering milestone lectures on the “Genetic basis of Congenital hypotrichosis” and “Molecular genetic progress of Alopecia Areata.”

I invite you to attend Hair India 2010, a maiden venture by the hair research society of India decorated by the invaluable contribution of the International and National luminaries in trichology, to be held on 3^rd^, 4^th^ and 5^th^ September this year in the ancient port city of Mamallapuram, an UNESCO world heritage site near Madras that is now Chennai. I am sure that the academic inputs in this excavation initiative by us to explore the hair follicle to uncover many mysterious treasures will be useful to mankind.

